# 17β-Estradiol (E_2_) Upregulates the ERα/SIRT1/PGC-1α Signaling Pathway and Protects Mitochondrial Function to Prevent Bilateral Oophorectomy (OVX)-Induced Nonalcoholic Fatty Liver Disease (NAFLD)

**DOI:** 10.3390/antiox12122100

**Published:** 2023-12-12

**Authors:** Ying Tian, Xinyu Hong, Yuan Xie, Zaixin Guo, Qi Yu

**Affiliations:** Department of Obstetrics and Gynecology, National Clinical Research Center for Obstetric & Gynecologic Diseases, Peking Union Medical College Hospital, Chinese Academy of Medical Sciences & Peking Union Medical College, Peking Union Medical College Hospital (Dongdan Campus), No.1 Shuaifuyuan Wangfujing Dongcheng District, Beijing 100730, China; yimuxu1224@163.com (Y.T.); hongxinyu@pumch.cn (X.H.); pumc_xieyuan@163.com (Y.X.); njbg111@126.com (Z.G.)

**Keywords:** surgical menopause, nonalcoholic fatty liver disease, menopausal hormone therapy, mitochondria, oxidative stress

## Abstract

Premature menopause is associated with an increased prevalence of nonalcoholic fatty liver disease (NAFLD). Menopausal hormone therapy (MHT) has been widely used in clinical practice and has the potential to protect mitochondrial function and alleviate NAFLD. After bilateral oophorectomy (OVX), female rats without 17β-estradiol (E_2_) intervention developed NAFLD, whereas E_2_ supplementation was effective in preventing NAFLD in female rats. The altered pathways and cellular events from both comparison pairs, namely, the OVX vs. sham group and the OVX vs. E_2_ group, were assessed using transcriptomic analysis. KEGG pathways enriched by both transcriptomic and metabolomic analyses strongly suggest that oxidative phosphorylation is a vital pathway that changes during the development of NAFLD and remains unchanged when E_2_ is applied. Liver tissue from the OVX-induced NAFLD group exhibited increased lipid peroxidation, impaired mitochondria, and downregulated ERα/SIRT1/PGC-1α expression. An in vitro study indicated that the protective effect of E_2_ treatment on hepatic steatosis could be abolished when ERα or SIRT1 was selectively inhibited. This damage was accompanied by reduced mitochondrial complex activity and increased lipid peroxidation. The current research indicates that E_2_ upregulates the ERα/SIRT1/PGC-1α signaling pathway and protects mitochondrial function to prevent OVX-induced NAFLD.

## 1. Introduction

Nonalcoholic fatty liver disease (NAFLD) is one of the leading chronic liver diseases worldwide, affecting approximately one-third of the world’s adult population and inflicting great health and economic burdens on all societies [1]. However, there are no specific treatments approved thus far. Potential treatments and research hotspots include some drugs approved for metabolic diseases, such as obesity and diabetes mellitus; intestinal microbiomes; herbal medicines; and lifestyle interventions [2]. Simple steatosis is not enough to trigger NAFLD. Multiple parallel factors, including oxidative stress, insulin resistance, inflammation, and mitochondrial dysfunction, contribute to the heterogeneity of NAFLD, which has led to the currently accepted “multiple-hit hypothesis” [3]. Mitochondrial dysfunction is a considerable factor in NAFLD, especially during the early stage of liver fat accumulation, fatty infiltration, and liver inflammation [4,5,6], and it is also a considerable factor that is closely associated with reproductive hormones [7].

Studies have suggested a strong relationship between sex and NAFLD [8]. Those factors that impact the time of reproductive hormone exposure, including age at menarche [9,10], parity, oral contraceptives, oophorectomy [11], and menopausal transition [12], are likely to impact the prevalence of NAFLD in females. Premature menopause is associated with an increased likelihood of having more severe fibrosis, while time from menopause is directly associated with an increased likelihood of having more severe fibrosis. The duration of estrogen deficiency in the postmenopausal state confers a risk of fibrosis among postmenopausal women with NAFLD [13]. Menopausal hormone therapy (MHT) is theoretically suitable in this situation, yet more mechanistic studies are needed to settle disputes regarding the exact benefits of MHT. Estrogen serves as a key regulator of energy homeostasis [14]. Females exhibit higher antioxidative capacity than males because female mitochondria exhibit higher antioxidant gene expression and lower oxidative damage [15]. MHT could maintain this ability and protect females even after menopause [16]. The effects of estrogen on mitochondria are very broad and involve dynamics [17] and function in the nervous system [18], skeletal muscle [19], bone [20], and liver [21]. The mechanism behind this strong regulation is largely unknown. 

Estrogen receptor alpha (ERα, ESR1) has been implicated in various liver diseases, including NAFLD, chronic hepatitis B (CHB), liver cirrhosis (LC) [22], and hepatocellular carcinoma (HCC) [23]. It was reported that dietary essential amino acids mediated metabolic restoration via ERα in the livers of ovariectomized (OVX) mice [24]. The classical estrogen signaling pathway through membrane ERα also reduces the lipid content in the liver [25]. Furthermore, the sex-specific response to a high-fat diet (HFD) through hepatic ERα explained the opposite consequences for hepatic health in males and females [26]. Based on the important role of estrogen and the potentially unique protective effect through hepatic ERα in the female group, we intended to explore the mechanism under this special guard. These efforts not only support MHT and provide lifelong protection for women, but also explore possible targets and related drugs contributing to those women in whom MHT is needed but not available, being limited to some special disease such as endometrial carcinoma and breast cancer.

Herein, we hypothesized that ERα contributed to the sex difference in NAFLD during MHT. We used OVX rats to induce NAFLD. Combined multi-omics analysis of liver tissue focused on mitochondrial function-related oxidative phosphorylation as a vital pathway regulated by 17-β-estradiol (E_2_). The impact of E_2_ on mitochondrial function though the ERα/SIRT1/PGC-1α signaling pathway was verified in vitro.

## 2. Materials and Methods

### 2.1. Animal and Study Design

The entire study protocol received approval from the Committee of Animal Experimentation (MDKN-2022-026). Female Sprague–Dawley (SD) rats (180 g–190 g) that were 8 weeks old were purchased from Beijing Vital River Laboratory Animal Technology Co., Ltd. (Beijing, China). All rats were raised under a specific pathogen-free (SPF)-grade environment and had free access to standard food and water. Before involvement in research, all rats were allowed one week for acclimation.

Anesthesia was induced by inhalation of isoflurane. The modeling surgery was performed through the back approach as previously reported [27]. After the estrous cycle was confirmed, rats in the OVX group underwent OVX, in which the bilateral ovaries and some surrounding adipose tissues were removed. Rats in the sham group underwent the same procedure, but only part of the adipose tissue around the ovaries was cut. 17β-Estradiol (E_2_, 20 μg/kg bw/day, E8875, Sigma, Livonia, MI, USA) or vehicle was subcutaneously applied to certain rats every day after the completion of surgery. All rats were checked daily, and their body weight was recorded weekly until sample collection.

Sample collection was conducted eight weeks after surgery. All animals went through fasting before sample collection. After anesthesia, blood samples were collected through an abdominal aorta puncture. After the completion of blood collection, overdose anesthesia of isoflurane was administered for euthanasia. Then, all other samples were collected, including liver tissue.

### 2.2. Cytological Examination of Vaginal Exfoliation

Two weeks of vaginal smears were applied to monitor the hormonal status and estrous cycle changes before and after surgery. As previously reported [28], wet cotton-tipped swabs were inserted into the vagina and rotated to obtain vaginal exfoliation. Then, the swabs were smeared evenly into a drop of saline on a slide. A Rapid Gram Stain Kit (BaSO, Zhuhai, China) was used, and cell morphology was evaluated under a microscope at 10× magnification [29]. Estrous cycle (proestrus, estrus, metestrus and diestrus) determination was based on cell types (karyocyte epithelial cells, keratinized epithelial cells and leukocytes). Exfoliated vaginal cells were dominated by karyocyte epithelial cells in the proestrus phase, by keratinized epithelial cells in the estrus phase, and by leukocytes in the diestrus phase, and consisted of approximately equal proportions of the above cell types in the metestrus phase.

### 2.3. Histopathological Analysis

Liver tissue samples were fixed in 4% paraformaldehyde (P1110, Solarbio, Beijing, China) at 4 °C for at least 24 h. After dehydration, paraffin immersion, and embedding, the samples were sectioned at a thickness of 4 μm. Hematoxylin and eosin (H&E) staining (G1120, Solarbio) was used for histological evaluation.

Fixed liver tissue was dehydrated in 15% and 30% sucrose solutions and embedded with an optimal cutting temperature compound (4583, Sakura, Tokyo, Japan). Samples were sectioned at a thickness of 8 μm. The Modified Oil Red O Stain Kit (G1261, Solarbio, Beijing, China) and Oil Red O Stain Kit For Cultured Cells (G1262, Solarbio, Beijing, China) were used to display hepatocyte steatosis in vivo and in vitro.

### 2.4. Immunohistochemistry (Paraffin) (IHC-P)

After dewaxing, the samples were subjected to routine dewaxing in water. Citrate buffer was used for antigen retrieval. IHC-P was performed using a 4-hydroxynonenal antibody (4-HNE, 1:300, MA5-27570, Invitrogen, Waltham, MA, USA) as the primary antibody. The subsequent procedures adhered to the instructions provided by Dako REAL EnVision Detection System, Peroxidase/DAB+, Rabbit/Mouse (K5007, DAKO, Agilent, Santa Clara, CA, USA).

### 2.5. Blood Biochemistry

Blood samples were collected and incubated for one night at 4 °C. Then, the samples were centrifuged at 3500 g/min for 20 min to obtain serum. Enzyme-linked immunosorbent assay (ELISA) was used to measure hormone levels, including E_2_ (E-OSEL-R0001, Elabscience, Wuhan, China), luteinizing hormone (LH, E-EL-R0026c, Elabscience), and follicle-stimulating hormone (FSH, E-EL-R0391c, Elabscience). Glutamic oxalacetic transaminase (AST), glutamic–pyruvic transaminase (ALT), gamma–glutamyl transpeptidase (GGT), alkaline phosphatase (ALP), total cholesterol (TC), triglycerides (TG), high-density lipoprotein (HDL), very low-density lipoprotein (vLDL), and low-density lipoprotein (LDL) were tested according to the kit instructions. The following kits were used: Aspartate Aminotransferase Kit (IFCC, BIOSINO, Beijing, China), Alanine Aminotransferase Kit (IFCC, BIOSINO, Beijing, China), Gamma-Glutamyl Transpeptidase Kit (Szasz, BIOSINO, Beijing, China), Alkaline Phosphatase Kit (IFCC, BIOSINO, Beijing, China), Cholesterol Kit (CHOD-PAP, BIOSINO, Beijing, China), Triglycerides Kit (GPO-PAP, BIOSINO, Beijing, China), Direct HDL–Cholesterol Kit (Clearance, BIOSINO, Beijing, China), Direct LDL–Cholesterol Kit (Clearance, BIOSINO, Beijing, China), and vLDL Lipoprotein Kit (Sino-uk bio, HY-N0033, Beijing, China).

### 2.6. Transmission Electron Microscopy (TEM)

Liver tissue was cut into 1 mm × 1 mm × 2 mm sections. Then, 2.5% glutaraldehyde (P1126, Solarbio, Beijing, China) and 1% osmic acid (18456, Ted Pella Inc., Redding, CA, USA) were used for prefixing and postfixing, respectively. After dehydration, immersion, and embedding, the samples were trimmed and cut into 70 nm sections using ultramicrotome (RMC, PT-PC). Double staining was performed with uranyl acetate and lead citrate. Then, the slices were ready for observation under TEM (HITACHI, HT7800, Tokyo, Japan).

### 2.7. Transcriptome Analysis

The total RNA of liver tissue was extracted using TRIzol (Invitrogen). The RNA integrity was assessed using the RNA Nano 6000 Assay Kit of the Bioanalyzer 2100 system (Agilent, Santa Clara, CA, USA), and libraries were prepared using the NEBNext^®^ Ultra™ RNA Library Prep Kit (Illumina, San Diego, CA, USA). After product purification on the AMPure XP system and library quality assessment on the Agilent Bioanalyzer 2100 system, libraries were pooled and sequenced for 150 bp paired-end sequencing on a NovaSeq 6000 (Illumina). The RNA-Seq data were converted to FASTQ format. Paired-end clean reads were aligned to the reference genome using Hisat2 v2.0.5. FeatureCounts v1.5.0-p3 was used to count the read numbers mapped to each gene. Then, the FPKM of each gene was calculated based on the length of the gene and read count mapped to this gene. Differential expression analysis was performed using the DESeq2 R package (1.20.0). Genes with an adjusted *p* value ≤ 0.05 and log2-fold change ≥1.0 were assigned as differentially expressed genes (DEGs). The ClusterProfiler R package was used for gene ontology (GO) and KEGG enrichment. GO enrichment was conducted according to cellular component (CC), molecular function (MF), and biological process (BP). R (4.3.0) software was used for further exploration.

### 2.8. Untargeted Metabolomics

Tissue samples were ground with liquid nitrogen into homogenates and resuspended in prechilled 80% methanol. After incubation and centrifugation, the supernatant was diluted with LC–MS-grade water and transferred to a fresh Eppendorf tube for recentrifugation. The raw data files generated by UHPLC–MS/MS were processed using Compound Discoverer 3.1 (CD3.1, Thermo Fisher, Waltham, MA, USA) to perform peak alignment, peak picking, and quantitation for each metabolite. Metabolite annotation was performed using the KEGG, HMDB, and LIPIDMaps databases. The metabolites with VIP > 1 and *p* value < 0.05 and fold change (FC) ≥ 2 or FC ≤ 0.5 were considered differentially expressed metabolites. R software was used for further exploration.

### 2.9. Western Blot (WB)

Liver tissue or cells were lysed in RIPA buffer (R0010, Solarbio) with PMSF (1:100, P0100, Solarbio) and protein phosphatase inhibitor (All-in-one, 100×) (1:100, P1260, Solarbio). Samples were centrifuged to obtain the protein supernatant. All protein samples were loaded in 5× SDS–PAGE loading buffer (P1040, Solarbio), heated in a 100 °C water bath for 10 min and stored. After sodium dodecyl sulfate–polyacrylamide gel electrophoresis (SDS–PAGE) and PVDF membrane transfer, membranes were blocked in 5% skim milk powder dissolved in TBST for 1 h at room temperature. Membranes were incubated with anti-estrogen receptor alpha antibody (1:500, ab32063, Abcam, Cambridge, UK) and anti-SIRT1 antibody (1:1000, ab189494, Abcam), anti-PGC1 alpha antibody (1:500, Ab191838, Abcam), phospho-ACC1 (p-ACC1, Ser79) antibody (1:1000, 29119-1-AP, Proteintech, Wuhan, China), ACC1 polyclonal antibody (1:2000, 21923-1-AP, Proteintech, Wuhan, China), and ACLY monoclonal antibody (1:2000, 67166-1-Ig, Proteintech) at room temperature for 2 h. After incubation with the corresponding secondary antibody, goat anti-rabbit IgG H&L (HRP) (1:20,000, ab97051, Abcam) SuperEnhanced chemiluminescence reagents (W0001-1, Applygen, Beijing, China) were applied to the membranes for final detection. Stripping solution (P1650, Applygen) was used to remove antibodies from membranes to allow anti-GAPDH antibody (1:5000, ab181602, Abcam, Cambridge, UK) incubation and detection.

### 2.10. Cell Lines and Treatment

The BRL 3A cell line (CL-0036, Pricella, Wuhan, China) was treated with vehicle, linoleic acid (LA, L1012, Sigma), E_2_, fulvestrant (HY-13636, Sigma), or selisistat (Ex527, HY-15452, Sigma). A TG concentration colorimetric assay kit (E-BC-K261-M, Elabscience) was used to evaluate hepatocyte steatosis in the cell line. Cell Counting Kit-8 (CCK-8, CK04, DoJinDo, Beijing, China) was used for cell death determination.

### 2.11. Measurement of Mitochondrial Complex Activity

The Mitochondrial Complex I Activity Assay Kit (E-BC-K149-M, Elabscience, Beijing, China), Mitochondrial Complex II Activity (E-BC-K150-M, Elabscience, Beijing, China), Mitochondrial Complex III Activity Colorimetric Assay (E-BC-K151-M, Elabscience, Beijing, China), Mitochondrial Complex IV Activity Assay Kit (E-BC-K152-M, Elabscience, Beijing, China), and Mitochondrial Respiratory Chain Complex V Activity (E-BC-K153-M, Elabscience, Beijing, China) were used to evaluate mitochondrial complex activity.

### 2.12. Lipid Peroxidation Assay

BODIPY™ 581/591 C11 (D3861, Invitrogen) was used to demonstrate lipid peroxidation. As described in the instructions, 1 mg of BODIPY™ 581/591 was dissolved in DMSO as a stock solution (10 mM) and diluted to generate the working solution (5 μM) with maintenance medium. The working solution was applied at 0.5 mL/well to cover the cells for 30 min at 37 °C in the dark. Images were photographed randomly under confocal fluorescence microscopy (TCS SP8) and analyzed using Image J (Java 1.8.0_322 (64 bit)).

### 2.13. Statistical Analysis

Data were collected and managed with Microsoft Excel software (16.0.17029.20028). For two-group and multiple-group comparisons, one-way ANOVA and unpaired t tests (two-tailed) were applied to evaluate significance using GraphPad Prism 9 software. Statistical significance was considered and expressed as * *p* < 0.05; ** *p* < 0.01; *** *p* < 0.001; and **** *p* < 0.0001.

## 3. Results

### 3.1. E_2_ Supplementation Alleviated OVX-Induced NAFLD in Female Rats

**Figure 1:**
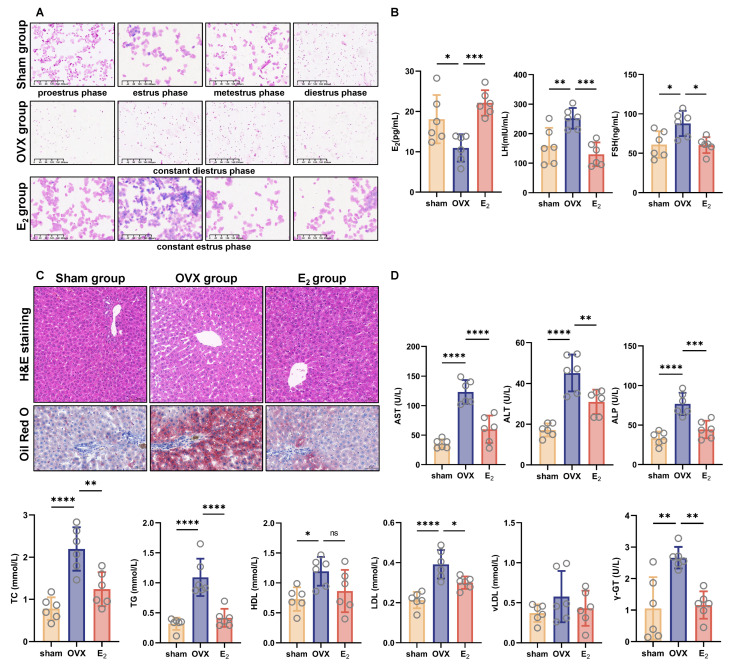
Female rats developed NAFLD after OVX, which was reversed by E_2_ supplementation. (**A**) Representative images of vaginal exfoliation staining in different estrous cycle stages (scale bar = 200 μm). (**B**) Serum levels of LH, FSH, and E_2_ (*n* = 6). (**C**) Representative images of oil red O staining and H&E staining of liver tissue (scale bar = 50 μm). (**D**) Serum levels of liver enzymes, blood lipids and lipoproteins. * *p* < 0.05; ** *p* < 0.01; *** *p* < 0.001; and **** *p* < 0.0001.

We performed OVX on female rats to induce NAFLD. Hormone levels gradually stabilized two weeks after surgery; that is, the OVX group exhibited only the diestrus phase, whereas the E_2_ group appeared to continuously be in the estrus phase and the control group went through a normal estrous cycle (Figure 1A). The decreased serum E_2_ levels and increased serum LH and FSH levels verified the existence of reproductive hormone deficiency, which could be alleviated by E_2_ supplementation (Figure 1B). Eight weeks after surgery, rats in the OVX group developed NAFLD as estimated by pathological analysis (Figure 1C). At the same time, serum biochemistry testing indicated that liver function was impaired because AST, ALT, ALP and GGT levels were increased in the OVX group and that blood lipids were abnormal because the levels of TC, TG and LDL were high in the OVX group (Figure 1D). E_2_ supplementation contributed to protecting liver function and preventing NAFLD development.

### 3.2. E_2_ Supplementation Maintained Some of the Pathways in Liver Tissue That Were Altered by OVX

**Figure 2:**
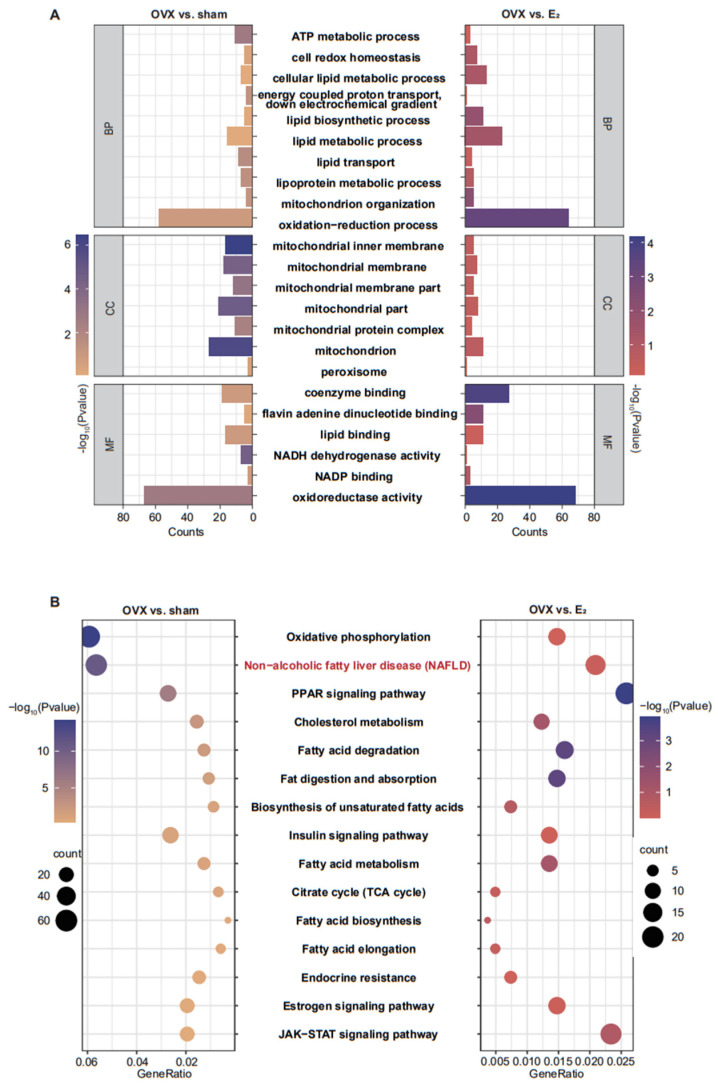
Transcriptomic analysis of liver tissue. (**A**) Double bar plot of the GO pathway enrichment analysis showing the shared pathways from the OVX group vs. E_2_ group and the OVX group vs. sham group. (**B**) Double dot plot of the KEGG pathway enrichment results showing the shared pathways from the OVX group vs. E_2_ group and the OVX group vs. sham group.

During the processes of NAFLD development in rats in response to reproductive hormone deficiency and in the protection against NAFLD afforded by the timely use of E_2_, transcriptomic analysis of liver tissue found that various pathways were altered. GO analysis demonstrated that the biological processes included oxidation−reduction process, lipid metabolic processes, and cell redox homeostasis; the cellular components included mitochondrion, mitochondrial protein complex and mitochondrial membrane; and the molecular functions included oxidoreductase activity, flavin adenine dinucleotide binding, and NADH dehydrogenase activity, which were highly enriched in the OVX vs. sham comparison and in the OVX vs. E_2_ comparison (Figure 2). KEGG analysis indicated that, compared with those in the sham group, various pathways in the OVX group were changed, including oxidative phosphorylation, NAFLD, cholesterol metabolism, fatty acid metabolism, and the estrogen signaling pathway. All of these changes in the functions of the liver were protected by E_2_ supplementation (Figure 2B).

### 3.3. The Combination of Transcriptomic and Metabolomic Analyses Revealed Enrichment of Oxidative Phosphorylation as a Key Pathway That Was Altered in the OVX-Induced NAFLD and E_2_ Supplementation Groups

**Figure 3:**
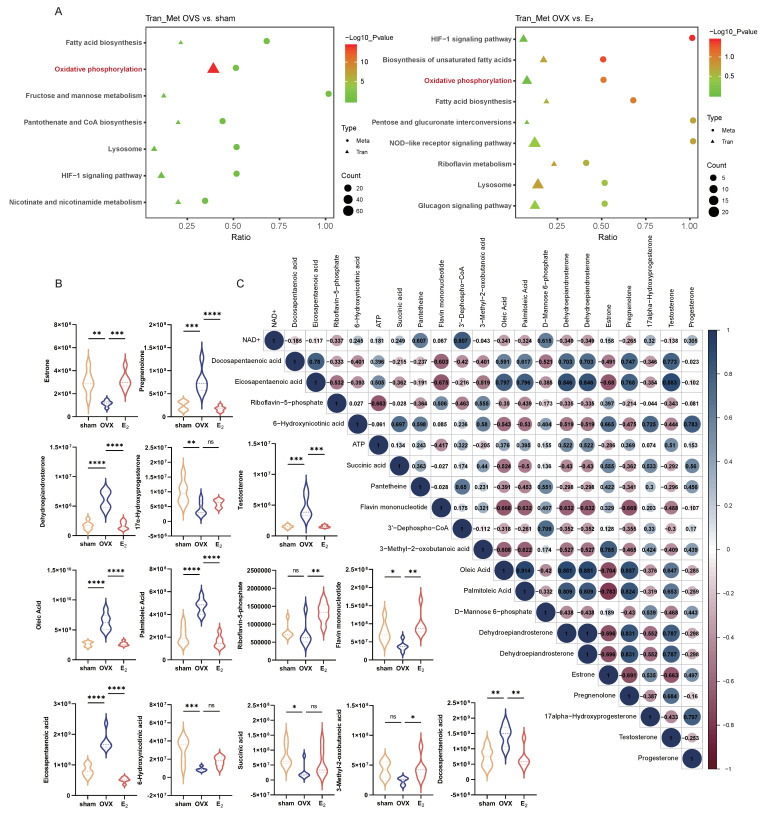
Metabonomic analysis of liver tissue. (**A**) Co-enrichment KEGG pathways of transcriptomic and metabonomic analyses in both comparisons. (**B**) Differentially expressed metabolites in the biosynthesis of the unsaturated fatty acid pathway, the oxidative phosphorylation pathway, and the ovarian steroidogenesis pathway. (**C**) Correlation heatmap of the differentially expressed metabolites. * *p* < 0.05; ** *p* < 0.01; *** *p* < 0.001; and **** *p* < 0.0001.

Both transcriptomic and metabolomic analyses of liver tissue from two comparisons (the OVX vs. sham group and the OVX vs. E_2_ group) focused on the oxidative phosphorylation pathway. Other pathways that were coenriched were fatty acid biosynthesis, the lysosome pathway, and the HIF-1 signaling pathway. In addition, in the OVX group, fructose and mannose metabolism, pantothenate and CoA biosynthesis, and nicotinate and nicotinamide metabolism were coenriched compared with the sham group, while biosynthesis of unsaturated fatty acids, the NOD-like receptor signaling pathway, riboflavin metabolism, and the glucagon signaling pathway were coenriched compared with the E_2_ group (Figure 3A). More specifically, the livers of mice with OVX-induced NAFLD exhibited decreased estrone and increased testosterone levels, while the other two groups exhibited the opposite trend in terms of altered reproductive hormone levels. Increased oleic acid, palmitoleic acid, eicosatetraenoic acid, and docosapentaenoic acid levels in the OVX group represented the changes in fatty acid biosynthesis that could be more or less be ameliorated by E_2_ supplementation. Metabolites involved in the oxidative phosphorylation pathway, including riboflavin-5-phosphate, flavin mononucleotide, and succinic acid, were present at abnormal levels in liver tissue from the OVX-induced NAFLD group and tended to improve when E_2_ was applied (Figure 3B). Correlation analysis of the relevant metabolites also demonstrated that reproductive hormone levels were closely related to fatty acid metabolism and mitochondrial functions (Figure 3C).

### 3.4. E_2_ Supplementation Upregulated the ERα/SIRT1 Pathway and Alleviated OVX-Induced NAFLD

**Figure 4:**
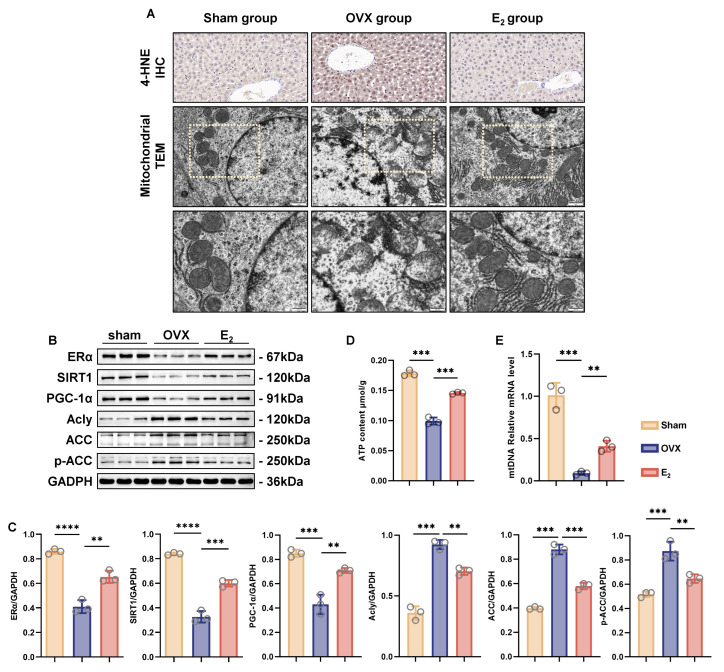
E_2_ supplementation protected against mitochondrial dysfunction caused by OVX in vivo. (**A**) Representative images of 4-HNE immunohistochemical staining (scale bar = 20 μm) and mitochondria in liver tissue under TEM (scale bar = 1 μm and 500 nm). (**B**,**C**) Representative images of WB and quantitative analysis showing the expression levels of ERα, SIRT1, PCG-1α, Acly, ACC, and p-ACC in liver tissue (*n* = 3). (**D**,**E**) Measurement of the ATP content and relative mtDNA level of liver tissue (*n* = 3). ** *p* < 0.01; *** *p* < 0.001; and **** *p* < 0.0001.

Given the potential for mitochondrial dysfunction to exist in the OVX-induced NAFLD group, we further evaluated the morphology and function of mitochondria. Excess 4-HNE suggested that lipid oxidation occurred in rats from the OVX group, which meant that there could be mitochondrial impairment. This damage could be observed under TEM (Figure 4A). Liver mitochondria from rats in the OVX group exhibited slight enlargement and swelling, accompanied by indistinct inner ridges. E_2_ supplementation prevented a certain degree of mitochondrial dysfunction and further prevented the development of NAFLD. Sirtuins are a family that plays an irreplaceable role in multiple metabolic disorders [30]. We evaluated the expression levels of ERα, the receptor of the classical estrogen signaling pathway, and SIRT1, which is involved in reducing oxidative stress in various diseases [31,32,33]. ERα and SIRT1 were decreased in the livers of the OVX-induced NAFLD group. E_2_ supplementation upregulated the expression of the ERα/SIRT1/PGC-1α pathway. In contrast, acetyl-CoA carboxylase (ACC), coordinating the synthesis and oxidation of fatty acids [34], and ATP citrate lyase (Acly), controlling flux through the de novo lipogenesis pathway in liver [35], were both increased in the OVX group, which was consistent with the occurrence of NAFLD. ACC is deactivated after phosphorylation. As expected, p-ACC was regulated in the same manner as ACC. These disturbances were all alleviated through E_2_ supplementation (Figure 4B,C). The decreased ATP content and relative mtDNA levels in the OVX group verified the impairment in mitochondria, which could be protected with E_2_ supplementation (Figure 4D,E).

### 3.5. E_2_ Treatment Alleviated LA-Induced Hepatic Steatosis by Upregulating the ERα/SIRT1 Pathway

**Figure 5:**
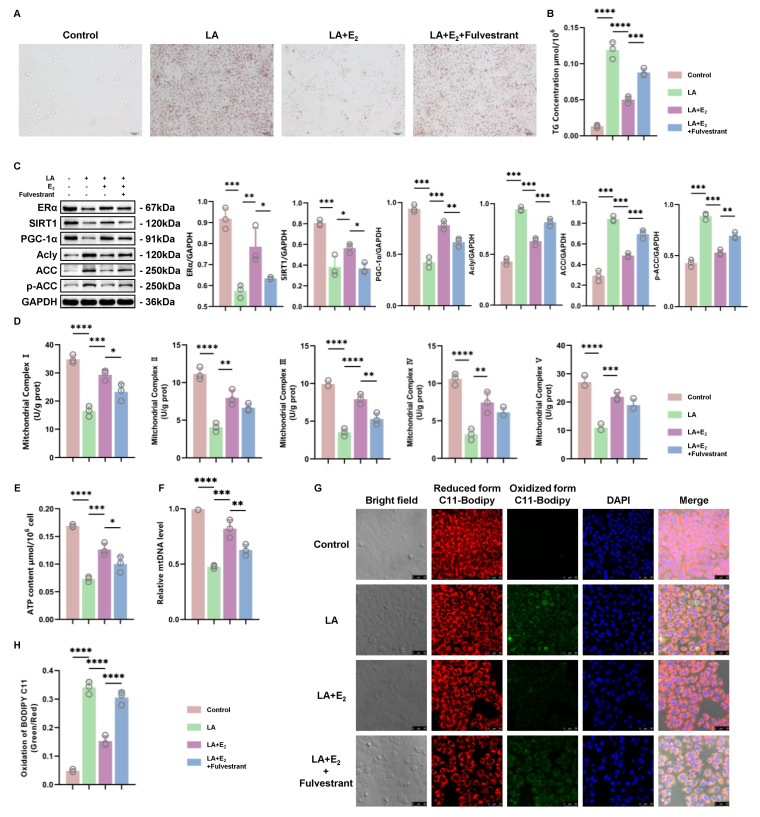
ERα inhibitor blocked the improvement effect of E_2_ on mitochondrial function and increased hepatocyte steatosis. (**A**) Representative views of oil red O staining of BRL 3A cells treated with LA (200 μM), E_2_ (0.1 μM), or fulvestrant (1 μM) for 48 h. (**B**) Measurement of the TG concentration in cells (*n* = 3). (**C**) Representative images of WB and quantitative analysis showing the expression levels of ERα, SIRT1, PCG-1α, Acly, ACC, and p-ACC in treated cells (*n* = 3). (**D**) Measurement of mitochondrial complex activity in treated cells (*n* = 3). (**E**,**F**) Measurement of the ATP content and relative mtDNA level of treated cells (*n* = 3). (**G**,**H**) Representative images of the BODIPY C11581/591 staining (scale bar = 50 μm) and quantitative analysis. The cell nuclei were stained with DAPI (blue). * *p* < 0.05; ** *p* < 0.01; *** *p* < 0.001; and **** *p* < 0.0001.

Then, we performed an in vitro study to explore the hypothesis that E_2_ treatment could protect mitochondrial function, reduce lipid oxidation, and prevent hepatic steatosis by upregulating the ERα/SIRT1/PGC-1α pathway. The LA-induced TG accumulation could be alleviated by E_2_ treatment, and this protection was abolished by the estrogen receptor inhibitor fulvestrant (Figure 5A,B). Fulvestrant inhibited E_2_ from upregulating the ERα/SIRT1/PGC-1α pathway (Figure 5C) and downregulating Acly, ACC, and p-ACC, subsequently abolishing the protective effects of E_2_ on mitochondria. The decreased ATP content, relative mtDNA levels, and mitochondrial complex activity verified the impairment in mitochondria (Figure 5D–F). Mitochondrial dysfunction induced by LA also caused increased lipid oxidation, which could be prevented by E_2_ treatment and worsened by fulvestrant (Figure 5G,H). These results indicated that E_2_ treatment could protect mitochondrial function and further protect hepatocytes from developing hepatic steatosis. This effect relied on the expression level of ERα because ERα inhibition abolished all of these benefits from E_2_ treatment.

**Figure 6:**
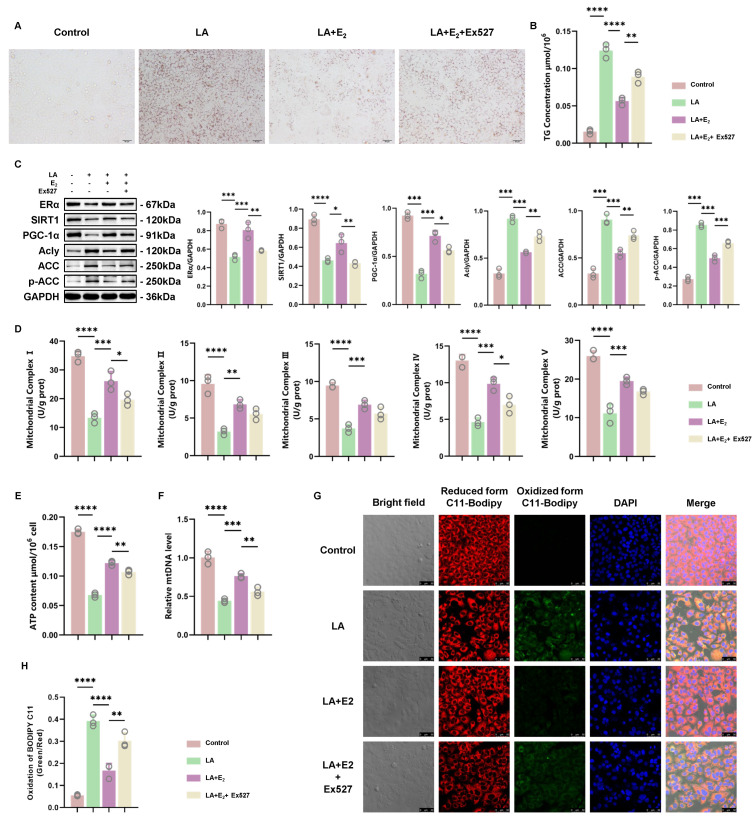
SIRT1 inhibitor blocked the improvement effect of E_2_ on mitochondrial function and increased hepatocyte steatosis. (**A**) Representative views of oil red O staining of BRL 3A cells treated with LA (200 μM), E_2_ (0.1 μM), or Ex527 (1 μM) for 48 h. (**B**) Measurement of the TG concentration in cells (*n* = 3). (**C**) Representative images of WB and quantitative analysis showing the expression levels of ERα, SIRT1, PCG-1α, Acly, ACC, and p-ACC in treated cells (*n* = 3). (**D**) Measurement of mitochondrial complex activity in treated cells (*n* = 3). (**E**,**F**) Measurement of the ATP content and relative mtDNA level of treated cells (*n* = 3). (**G**,**H**) Representative images of BODIPY C11581/591 staining (scale bar = 50 μm) and quantitative analysis. The cell nuclei were stained with DAPI (blue). * *p* < 0.05; ** *p* < 0.01; *** *p* < 0.001; and **** *p* < 0.0001.

Then, we found that selectively inhibiting SIRT1 also abolished the effects of E_2_. That is, Ex527 caused excess TG accumulation even when E_2_ was used (Figure 6A,B). This could be explained by Ex527 inhibiting E_2_ from upregulating ERα/SIRT1/PCG-1α expression. The reemergence of elevated Acly, ACC, and p-ACC expression suggested a loss of proper regulation in hepatic lipid metabolism. (Figure 6C). Inhibition of SIRT1 by Ex527 also caused a decrease in the ATP content, relative mtDNA level, and mitochondrial complex activity and increased lipid oxidation, abolishing the E_2_ benefits preventing hepatic steatosis (Figure 6D–H). In summary, an in vitro study indicated that E_2_ treatment alleviated LA-induced hepatic steatosis by upregulating the ERα/SIRT1/PCG-1α pathway.

## 4. Discussion

In this study, ovariectomized female rats were used to induce hormone deficiency-related NAFLD. We subcutaneously applied E_2_, alleviating fat deposition in the liver. KEGG enrichment of both the transcriptomics and metabolomics of liver tissue highlighted that oxidative phosphorylation was dysregulated during OVX-induced NAFLD. Furthermore, the ERα/SIRT1/PCG-1α expression level was decreased, which was related to mitochondrial impairment and lipid peroxidation. Accordingly, expression levels of Acly, ACC, and p-ACC were upregulated in the OVX group. An in vitro study using BRL 3A cells indicated that E_2_ treatment was able to alleviate LA-induced hepatic steatosis, protect mitochondrial function, and mitigate lipid peroxidation. These benefits were abolished when fulvestrant and Ex527 were added. The results indicated that E_2_ protected female rats from the development of NAFLD after OVX by upregulating the ERα/SIRT1/PCG-1α signaling pathway.

During the first decade after menopause, females experience dramatic changes in their physiological state, including increased weight gain and obesity, and a consequent increased prevalence of metabolic syndrome, diabetes, osteoporosis, cardiovascular disease and dementia [36]. Menopause at an early age or surgical menopause before natural menopausal age results in a longer exposure to hormone deficiency. Therefore, the occurrence of estrogen withdrawal heralds an important opportunity for early intervention. Previous data showed that MHT, especially estrogen, contributed to a consistently decreased mortality with a favorable risk–benefit profile in low-risk women whose menopause-related hormone deficiency occurred at age 60 [36]. The hypoestrogenic state appears to be an endocrine hit on the development of NAFLD. Estrogens are effective in hindering the progression of NAFLD due to their contribution to reducing inflammation, improving mitochondrial function, and alleviating oxidative stress and insulin resistance [37]. The mechanism behind this protective effect is complicated. It was reported that E_2_ was involved in improving mitochondrial function by inducing JNK activation to mitigate fatty acid–insulin resistance in hepatocytes [38]. PGC1β, a novel hepatic protective factor, is modulated by E_2_, contributing to the promotion of mitochondrial biogenesis and function [39]. PGC1α also participates in the sex-dependent modulation of liver mitochondria and oxidative stress during the development of NAFLD induced by a HFD [21,40]. The signaling process between E_2_ and downstream molecules is unknown. Pathway preferential estrogen 1 (PaPE-1) is a novel estrogen receptor ligand that has been shown to favorably affect metabolic functions [41]. Estrogen-related receptor alpha (ERRα) was reported to contribute to the sex disparity in NAFLD development by regulating hepatic triglyceride biosynthesis and VLDL assembly and secretion, providing a novel treatment target [42,43]. Furthermore, ERα, as reported before, was nonnegligible during the development of OVX-induced NAFLD [24,25,44]. Our data highlighted the importance of E_2_ in protecting mitochondrial function, reducing lipid deposition, and preventing NAFLD. We proved that E_2_ supplementation prevented NAFLD induced by OVX in female rats by upregulating ERα/SIRT1 expression levels. Future studies may consider the development of novel drugs targeting ERα for both males and females to prevent NAFLD [26].

As a group of NAD+-dependent III deacetylases, the sirtuin (SIRT1-7) family plays an important role in regulating mitochondrial biogenesis and participates in the progression of NAFLD [45]. Elevating Sirt1 levels and activating the AMPK pathway protect hepatocytes from lipid metabolic disorders such as NAFLD and suppress lipogenesis [46]. Restoration of hepatic SIRT2 expression in HFD-fed mice largely alleviated hepatic steatosis, whereas SIRT2 liver-specific ablation exacerbated these metabolic dysfunctions [47]. Sirt3 overexpression protected hepatocytes against mitochondrial apoptosis via the ERK-CREB signaling pathway [48]. Upregulated Sirt6 expression inhibited HFD-induced hepatic steatosis, insulin resistance, and inflammation [49]. Our research suggested that Sirt1 could serve as a bridge through which E_2_ regulates mitochondrial function in NAFLD. Sirt1 has been a focal point of mitochondrial dysfunction in NAFLD for some time. TM4SF5-mediated SIRT1 modulation was dysregulated in steatotic or steatohepatitic liver tissue from high-fat diet or CCl4-treated mice and human patients [50]. The SIRT1/AMPK signaling pathway was noted in several experiments that involved treatments with plant ingredients, such as phloretin [51], 11β-HSD1 inhibitor [52], berbamine [53], and pterostilbene [54]. IGF2 knockdown reduced the MMP level and increased the production of reactive oxygen species (ROS) by suppressing downstream SIRT1/PGC1α expression in NAFLD [55]. Our research, for the first time, reported that Sirt1 participated in preventing OVX-related NAFLD downstream of Erα under the regulation of E_2_. The Sirt1 inhibitor Ex527 blocked the protective effect of E_2_ on decreasing TG accumulation, relieving lipid peroxidation and mitigating LA-induced hepatic steatosis. Exploration of Sirt1 as a vital downstream target of Erα also provides a potential target, particularly when E_2_ supplementation is limited due to the clinical state. SIRT1 functioning in both males and females is worth exploring for its translational application and expansion of indications.

In both clinical and basic research, MHT, primarily estrogens, was considered effective in preventing NAFLD caused by hormone deficiency. There has been much controversy about MHT, including the timing, duration, and dosage of the intervention [56]. Early-onset MHT prevents oxidative stress and metabolic alterations caused by ovariectomy [57], while it seems ineffective if MHT is applied too late. That is, there is a so-called “window of opportunity” [58]. This intervention window emphasizes that except for revealing the mechanism behind the effectivity of E_2_ in hormone deficiency-induced NAFLD, uncovering the mechanism for why the “window of opportunity” exists is also vital. Mitochondrial dysfunction arouses wide concern in MAFLD-related studies, as reported in our research and previous studies. Hepatocyte mitophagy and aggravation of mitochondrial damage were observed in a fructose-induced nonalcoholic steatohepatitis (NASH model and could be inhibited by the TNFα/Miz1-positive feedback loop [59]. Although NAFLD might improve after some treatments, it doesn’t improve completely. Mitochondrial impairment existed persistently after the restoration of steatosis in hepatocytes, partly due to early adaptative respiratory increase, which indicated the persistence of potential health risks associated with reversing NAFLD in patients [60]. That is, prevention means much more than reducing the burden of disease and improving quality of life. MHT has the potential to prevent the occurrence of NAFLD and is, to a great extent, more natural and safer for women who have undergone menopause or surgical menopause at an early age. In addition, sex differences, menopausal status, age, and other reproductive information in clinical follow-up and gene association studies of NAFLD should be taken into full consideration to fill current gaps and choose applicable treatment for females with NAFLD [61].

## 5. Conclusions

In conclusion, E_2_ supplementation prevented OVX-induced NAFLD in female rats by upregulating the ERα/SIRT1/PCG-1α signaling pathway and protecting mitochondrial function.

## Data Availability

The datasets used and/or analysed during the current study are available from the corresponding author on reasonable request.
